# Influence of the Molar Activity of ^203/212^Pb-PSC-PEG_2_-TOC on Somatostatin Receptor Type 2-Binding and Cell Uptake

**DOI:** 10.3390/ph16111605

**Published:** 2023-11-14

**Authors:** Marc Pretze, Enrico Michler, Roswitha Runge, Kerstin Wetzig, Katja Tietze, Florian Brandt, Michael K. Schultz, Jörg Kotzerke

**Affiliations:** 1 Department of Nuclear Medicine, University Hospital Carl Gustav Carus, Technical University Dresden, Fetscherstr. 74, 01307 Dresden, Germany; enrico.michler@ukdd.de (E.M.); roswitha.runge@ukdd.de (R.R.); kerstin.wetzig@ukdd.de (K.W.); katja.tietze@ukdd.de (K.T.); florian.brandt@ukdd.de (F.B.); joerg.kotzerke@ukdd.de (J.K.); 2 Department of Radiology, University of Iowa, Iowa City, IA 52240, USA; mschultz@perspectivetherapeutics.com; 3 Viewpoint Molecular Targeting, Inc. (DBA Perspective Therapeutics), Coralville, IA 52241, USA; 4 Department of Chemistry, University of Iowa, Iowa City, IA 52241, USA; 5 Department of Radiation Oncology, University of Iowa, Iowa City, IA 52242, USA

**Keywords:** ^203^Pb, ^212^Pb, TOC, cell uptake, AR42 J, molar activity (A_M_), neuroendocrine tumor (NET), somatostatin receptor (SST2), targeted alpha-therapy (TAT)

## Abstract

(1) Background: In neuroendocrine tumors (NETs), somatostatin receptor subtype 2 is highly expressed, which can be targeted by a radioactive ligand such as [^177^Lu]Lu-1,4,7,10-tetraazacyclododecane-*N*,*N*′,*N*″,*N*‴-tetraacetic acid-[Tyr^3^,Thr^8^]-octreotide (^177^Lu-DOTA-TOC) and, more recently, by a lead specific chelator (PSC) containing ^203/212^Pb-PSC-PEG_2_-TOC (PSC-TOC). The molar activity (A_M_) can play a crucial role in tumor uptake, especially in receptor-mediated uptake, such as in NETs. Therefore, an investigation of the influence of different molar activities of ^203/212^Pb-PSC-TOC on cell uptake was investigated. (2) Methods: Optimized radiolabeling of ^203/212^Pb-PSC-TOC was performed with 50 µg of precursor in a NaAc/AcOH buffer at pH 5.3–5.5 within 15–45 min at 95° C. Cell uptake was studied in AR42 J, HEK293 sst2, and ZR75-1 cells. (3) Results: ^203/212^Pb-PSC-TOC was radiolabeled with high radiochemical purity >95% and high radiochemical yield >95%, with A_M_ ranging from 0.2 to 61.6 MBq/nmol. The cell uptake of ^203^Pb-PSC-TOC (A_M_ = 38 MBq/nmol) was highest in AR42 J (17.9%), moderate in HEK293 sstr (9.1%) and lowest in ZR75-1 (0.6%). Cell uptake increased with the level of A_M_. (4) Conclusions: A moderate A_M_ of 15–40 MBq/nmol showed the highest cell uptake. No uptake limitation was found in the first 24–48 h. Further escalation experiments with even higher A_M_ should be performed in the future. It was shown that A_M_ plays an important role because of its direct dependence on the cellular uptake levels, possibly due to less receptor saturation with non-radioactive ligands at higher A_M_.

## 1. Introduction

Recently, receptor-targeted α-therapy (TAT) has gained importance in nuclear medicine clinical routine, especially for tumor patients who develop resistance to β^–^-therapy [1,2]. Typically, patients receive multiple doses of ^90^Y or ^177^Lu (dose of 5–8 GBq per patient) at periodic intervals of administration (e.g., 8-week intervals) [3]. Unfortunately, a significant proportion of these patients will eventually experience progressive disease and discontinue therapy. On the other hand, it has been observed that further responses and prolonged survival can be achieved by initiating α-therapy after disease progression. For example, the use of ^225^Ac (dose: 100 kBq/kg, four α-particles per decay) can dramatically reduce the required level of administered radioactivity (by a factor of about 1000 compared to ^177^Lu). However, the behavior of α-particles in tumor cells is complicated by α-emitting radionuclide progeny in the ^225^Ac series. Of particular interest are ^221^Fr, ^217^At, ^213^Bi, and ^213^Po. A key issue is the biological fate of ^213^Bi (t_1/2_ 46 min), which is transported out of the tumor cells (by its own escape or by the escape of ^221^Fr or ^217^At) and accumulates mainly in the kidneys, delivering an α-emitting dose of ^213^Po, which, in turn, could have a higher negative impact on renal function [4]. Therefore, it is often suggested that only patients with an efficient renal function can be considered eligible for ^225^Ac therapy, while patients with impaired renal function may not be eligible for these therapies.

^212^Pb is a promising radionuclide for targeted alpha particle therapy that emits only one α-particle per β^–^-decay of ^212^Pb to ^212^Bi and then either 64% to ^212^Po or 36% to ^208^Tl [5,6]. Therefore, a ^212^Pb-labeled radiopharmaceutical, once accumulated in the tumor tissue, will deposit its highest dose in the form of the α-particle specifically in the tumor cells, with a lower probability of further α-decay occurring in healthy organs. Thus, ^212^Pb represents a more favorable choice for cancer α-therapy patients who are naïve to (or who have progressed on) β^–^-therapy, including patients with reduced renal function. Ongoing preclinical and clinical studies are investigating the potential of ^212^Pb-labeled peptides and antibodies (at a dose of approximately 2 MBq/kg) [4] or approximately between ^177^Lu and ^225^Ac administered doses of radioactivity. Lead specific chelator-PEG_2_-[Tyr^3^,Thr^8^]-octreotide (PSC-PEG_2_-TOC) (VMT-α-NET) is a somatostatin subtype 2 (SST2) receptor targeting peptide for the treatment of neuroendocrine tumors (NETs) that exhibits rapid tumor accumulation, high tumor retention, and rapid renal excretion [7]. It carries the chelator PSC [7], which forms highly stable complexes with ^203/212^Pb and, in contrast to less stable 1,4,7,10-tetraazacyclododecane-*N*,*N*′,*N*″,*N*‴-tetraacetic acid (DOTA) complexes, remains intact even after β^–^ conversion to ^212^Bi [8]. Recently, the true matched pair ^203/212^Pb has come into focus through several first in-human theranostic applications [9,10,11]. While ^203^Pb (t_1/2_ = 52 h; 279 keV gamma ray; 81% intensity) represents an ideal elementally matched imaging surrogate, ^212^Pb itself can be used for SPECT imaging [12]. A true matched pair could finally overcome the differential pharmacokinetic/pharmacological properties observed between diagnostic and therapeutic radiotracers with unmatched radionuclides pairs [13].

In addition to somatostatin analogs, such as PSC-TOC, other radiopharmaceuticals targeting other receptors (e.g., PSMA derivatives) for radiolabeling with ^203/212^Pb are under preclinical investigation and may soon be translated into clinical use [4,14,15,16]. Emerging evidence suggests that α-particles have the potential to overcome resistance to β^–^-therapy and could lead to further therapeutic options for patients with palliative effects and, in some cases, even complete remission [17]. Furthermore, the appearance of Cherenkov light due to the decay of ^212^Pb may be useful not only for diagnostics but also for further treatment options [18]. The optimal mass of the precursor peptide for radiopharmaceuticals is an important parameter in radiopharmaceutical development to ensure that the highest degree of tumor targeting with the lowest accumulation and retention in normal organs and healthy tissues is achieved. This parameter is also important in achieving a formulation that results in smooth radiometallation and a high radiochemical yield and purity. In general, this parameter is known as the molar activity (A_M_), which plays a crucial role for all PET radiotracers targeting saturable binding sites (e.g., receptors) but is secondary or negligible for many metabolic PET radiotracers, where the endogenous levels of the compound are in great excess of the radiotracer itself due to saturation of the receptors at the tumor site [19].

In this work, the influence of the A_M_-to-cell uptake of ^203/212^Pb-PSC-TOC was investigated in different cell lines (AR42 J, HEK293 sst2, and ZR75-1) to develop a more detailed understanding of the tracer in preparation for clinical use.

## 2. Results

### 2.1. Radiochemistry

The radiochemical yield (RCY) was 74–97% for ^203^Pb-PSC-TOC (Table 1) and 22–99% for ^212^Pb-PSC-TOC, depending on the reaction conditions (Table 2 and Table 3). The radiochemical purity (RCP) was always >95%. Stabilities greater than 95% of the formulated ^203/212^Pb-PSC-TOC were found up to 16 h at r.t. and for ^203^Pb-PSC-TOC up to 11 d at 4 °C by RP-HPLC. For ^212^Pb-PSC-TOC, HPLC is very useful for determining the identity of the product but not for the direct determination of RCY, according to the approach used here. A free ^208^Tl daughter nuclide was observed up to 20% in the chromatogram 4 h after radiosynthesis. Isothermal titration calorimetry (ITC) experiments confirmed the stability of the ^212^Bi daughter radionuclide in the PSC chelator to >95% at 2 h after radiolabeling. Importantly, the contribution of free ^208^Tl to the total body dose was shown to be negligible [20].

The optimization of the radiochemical labeling procedure included steps to overcome the loss of activity due to several purification steps: The freshly eluted activity from the ^224^Ra/^212^Pb generator is partitioned into Pb resin 10–12%, waste ~2%, and product 84–86%. The purification of ^212^Pb by Pb resin removes potential traces of the parent nuclide ^224^Ra, as well as the daughter nuclides ^212^Bi and ^208^Tl [21]. Therefore, activity measurements with a dose calibrator (ISOMED 2010, Nuvia Instruments GmbH, Dresden, Germany) in equilibrium of the mother and daughter nuclides show different values before and after Pb resin purification. Therefore, a normalization factor for the dose calibrator is required immediately after purification (Table 4). Further purification with C18 or Maxi-Clean (MC) SPE cartridges results in a further loss of product activity. The C18 contained 6–10% of the product activity, and the MC cartridge contained up to 40% of the product activity. It appears that the chelated ^212^Pb-PSC complex has strong binding competition with the sorbent material of the MC cartridges. Therefore, these cartridges are not suitable for purification. However, the cartridge purifications were later found to be obsolete due to further development of optimized radiolabeling conditions.

### 2.2. Cell Uptake Studies with Different Molar Activities

During the studies with ^203^Pb-PSC-TOC, it was found that SST2-transfected HEK293 cells showed a moderate cell uptake compared to endogenously SST2-positive AR42J cells, which showed a higher cell uptake. Compared to the routinely used ^68^Ga-DOTA-[Tyr^3^,Thr^8^]-octreotate (TATE) with a cell uptake in AR42J of about 8.7 ± 0.4% after 1 h, the uptake of ^203/212^Pb-PSC-TOC reached a comparable level 24 h after incubation only at the highest A_M_ > 7.9 MBq/nmol (^203^Pb-PSC-TOC) and A_M_ > 3.3 MBq/nmol (^212^Pb-PSC-TOC). ZR75-1 cells were used as a negative control and showed no significant uptake for all radiotracers used in this work.

It was found that cell uptake was strongly influenced by the number of cells seeded 1–2 days prior to the uptake experiments. With 100,000 cells seeded, very low cell uptake was observed, and the cell uptake increased with the number of cells seeded. The optimal number of cells for uptake was 1.0 million cells per well. Seeding cells three days prior to uptake had no further effect on cell uptake.

The optimum activity of ^203^Pb-PSC-TOC for incubation was found to be 100 kBq per well on a six-well plate, and cell viability was good for over 72 h. For ^212^Pb-PSC-TOC, activities greater than 50 kBq per well lead to a decrease in cell uptake and even lower cell viability (11% viability after 72 h), which affected the credibility of the cell uptake values. Therefore, activities of 25 kBq per well were used for ^212^Pb-PSC-TOC.

**Figure 1:**
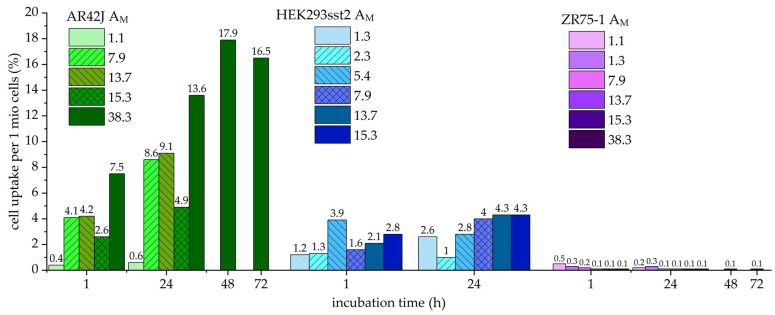
The cell uptake of ^203^Pb-PSC-TOC in different cell lines is shown. The values above the columns represent the relative mean cell uptake in percent. A_M_ is given in MBq/nmol. Each column represents the mean value of a triplicate. Shades of green: AR42J; shades of blue: HEK293 sst2; shades of purple: ZR75-1.

**Figure 2:**
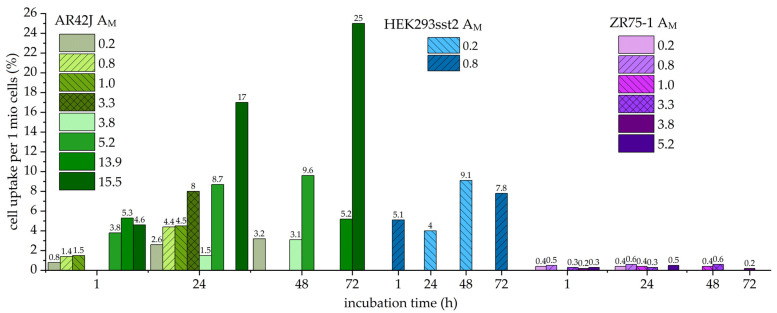
Cell uptake of ^212^Pb-PSC-TOC in different cell lines is shown. The values above the columns represent the relative mean cell uptake in percent. A_M_ is given in MBq/nmol. Each column represents the mean value of a triplicate. Shades of green: AR42J; shades of blue: HEK293 sst2; shades of purple: ZR75-1.

Figure 1 shows the cell uptake for ^203^Pb-PSC-TOC, and Figure 2 shows the cell uptake for ^212^Pb-PSC-TOC. It was found that the uptake increased with the A_M_ for both radioligands. In most of the experiments, only timeframes between 1 and 24 h were considered, because, for diagnostic purposes, patients are measured only within a timeframe of 24 h, and, for therapeutic purposes, most of the dose is already deposited (two half-lives = 75% of the dose, t_1/2_ = 10.64 h). In the case of the highest produced A_M_ of ^203/212^Pb-PSC-TOC, the timeframe was extended to 72 h in order to find a possible plateau or decrease in cell uptake. ^203^Pb-PSC-TOC showed an increase of relative cell uptake per 1 million cells up to 24.0 ± 0.8% even 149 h after incubation (2.9 × t_1/2_ of ^203^Pb). For ^212^Pb-PSC-TOC, the cell uptake still increased up to 25.0 ± 0.5% 72 h after incubation. Increased uptake values were found at the lowest excess of cold peptide (25%). These results could support the hypothesis that free binding sites of SST2 receptors are still available (Figure 2).

Table 5 and Table 6 show the statistical evaluation of the cell accumulation of ^203/212^Pb-PSC-TOC. Each cell line was incubated with the respective A_M_ in triplicate. The standard deviation of the mean accumulation was less than 10%, confirming that the data were consistent with the conclusions. The standard deviation for ZR75-1 uptake was not shown, because it was less than 0.04% in each experiment.

## 3. Discussion

### 3.1. Radiochemistry

Radiolabeling of PSC-TOC with ^203^Pb proceeded smoothly under standard labeling conditions. Challenges were observed with ^212^Pb when using the same conditions as for ^203^Pb that needed to be overcome. However, the addition of a higher mass of peptide precursor and slightly longer reaction times solved this problem [21]. The quality control showed an RCY >95% in most cases for ^203/212^Pb-PSC-TOC. It is noteworthy that, when the RCP for ^212^Pb-PSC-TOC was only >90%, this was due to ejected daughter nuclides that transferred to a solvent form [22]. When the developed TLC was remeasured after 2 h, the RCP increased to >95% (24 h later, the RCP was even >99%), as the free daughter nuclides decayed on the TLC within this time, confirming the radiochemical purity of ^212^Pb-PSC-TOC.

### 3.2. Influence of Molar Activity on Cell Uptake

For the cell uptake experiments performed in this work, it was observed that, the higher the A_M,_ the higher the cell uptake. An uptake limit was not found in these experiments, but there may be one, as can be found in the literature [19]. Furthermore, neither PSMA nor TATE peptides reached tumor saturation in the patients [23]. AR42J cells always showed the highest cell uptake for each A_M_ compared to HEK293 sst2 cells, which showed lower cell uptake. ZR75-1 cells as a negative control did not show significant cell uptake due to the lack of SST2 receptor. Therefore, AR42J cells may have the highest SST2 receptor expression of the cells used in this study. For further studies in this direction, transfected cells with even higher SST2 receptor expression than in AR42J cells could be used [24].

The high uptake of SST2-specific radioligands has been well examined for agonists and antagonists. It is known that agonists like TOC effectuate the internalization of SST2 receptors after stimulation in vivo and that this is the reason for the high and long-lasting uptake of SST2 radioligands [25]. However, this high accumulation seems to reduce after several treatments with β^–^-radioligands, due to differentiation of the SST2 expressing tumor cells. With only low SST2 expression at the tumor site, this could be one reason for the “β^–^ resistance” when only low amounts of radioligands reach the tumor site. The lower expression of SST2 receptor may merely be overcome by TAT, which seem to have a similar impact in tumor therapy at much lower doses compared to β^–^-therapy [26]. Therefore, antagonists like JR11, which have a similar accumulation to the agonistic radioligands, even after several treatments, may be a solution for this issue, since the SST2 expressing tumors do not differentiate when blocked by antagonists [27]. Another instrument to overcome the lowering SST2-specific tumor accumulation may be epigenetic stimulation before treatment or during a series of treatments. Experimental data have shown higher uptake rates in HEK293 sst2 cells up to 28 times versus untreated cells [28].

In radioligand therapy, a medium A_M_ tracer could reduce radiation exposure to dose-limiting organs with only a limited effect on radionuclide accumulation in the tumor. In this case, high A_M_ is considered to be 200 MBq/nmol, and low A_M_ is considered to be 2 MBq/nmol. Therefore, it can be assumed that the moderate A_M_ level of 15–40 MBq/nmol (Table 1 and Table 3) achieved in this work may be the optimal amount for application. Nevertheless, further cell studies with even higher A_M_ should be performed to confirm this. One way to achieve higher A_M_ is HPLC purification, which has been shown to increase the A_M_ of a ^68^Ga ligand by a factor of 10,000 [29]. In addition, a pilot therapy study with the optimal A_M_ can be performed against low A_M_ and high A_M_ to validate the observations from the cell studies. This experimental comparison may provide evidence to support the idea that moderate molar activities may indeed provide superior tumor uptake while minimizing radiation exposure to dose-limiting organs.

The amount of somatostatin analog administered varies with the analog used and the intended purpose of the administered drug. The TATE analog is administered at a high tracer level (100 nmol) in α- and β^–^-therapy, which is in the range of physiologically applied concentrations. This could lead to a lower tumor uptake if tumor receptors are blocked with an unlabeled peptide. Conversely, a higher A_M_ could negatively affect the tumor-to-organ ratio by saturating the physiologically expressing organs. For ^212^Pb therapy, this would mean that A_M_ = 1 MBq/nmol is closer to the amount of, e.g., Luthathera (50 MBq/nmol, 148 nmol for 7.4 GBq [30]) than A_M_ = 10 MBq/nmol.

A comparable study on the influence of the A_M_ applied with ^68^Ga or ^177^Lu was not found in the scientific literature. The results of an Al^18^F-labeled PSMA-11 study were presented [31], where it was found that the administration of a high A_M_ tracer increased the detection of low expression tumors while also increasing uptake in PSMA-expressing tissues, potentially leading to false-positive findings.

Taken together, variations in A_M_ may have different implications for diagnostic and therapeutic use. However, the moderate A_M_ of 15–40 MBq/nmol investigated in this work showed the highest cell uptake and should be used when administered to patients. For patients, a lower amount of peptide could increase tumor uptake while decreasing side effects.

## 4. Materials and Methods

All reagents and solvents were purchased from commercial suppliers at the highest purity and used without further purification. PSC-PEG_2_-TOC (VMT-α-NET) and ^224^Ra/^212^Pb generator (VMT-α-GEN) were obtained from Perspective Therapeutics Inc., Coralville, IA, USA. ^203^Pb solution in 8 M HCl was obtained from Cross Cancer Institute, Edmonton, AB, Canada. RCP was monitored by thin-layer chromatography (TLC) on iTLC-SG plates (Agilent, Santa Clara, CA, USA). Measurement of the radionuclide purity (RNP) and evaluation of the radio-TLC was performed with a thin-layer scanner (MiniScanPRO+, Eckert&Ziegler Eurotope GmbH, Berlin, Germany) equipped with a Model 43-2 alpha detector ZnS(Ag) scintillator (Ludlum Measurements, Sweetwater, TX, USA) and a build-in multi-channel analyzer (MCA) for gamma spectroscopy. Radio-HPLC was performed on a Shimadzu HPLC system (Thermo Scientific, Dreieich, Germany), equipped with a reverse-phase column (Merck Chromolith RP-18e; 100 × 4.6 mm plus a 5 × 4.6 mm guard column, Darmstadt, Germany) and a UV diode array detector (220 nm). The solvent system used was a gradient of acetonitrile:water (containing 0.05% TFA) (0–13 min: 0–60% MeCN) at a flow rate of 1.6 mL/min, unless otherwise stated. The pH was measured using a reflectance photometer (QUANTOFIX Relax, Macherey-Nagel GmbH & Co. KG, Düren, Germany).

### 4.1. Radiochemistry

Radiolabeling of the DOTA and PSC conjugates was performed according to standard protocols for these chelators [7]. Briefly, 50 µg precursor (DOTA-TATE (M = 1435.6 g/mol) or PSC-PEG_2_-TOC (PSC-PEG_2_-TOC, M = 1578.7 g/mol)) in H_2_ O_suprapure_ was added to a 10 mL reaction vial together with 100 µL EtOH_absolute_, 290 µL 1 M NaAc/AcOH buffer (pH 4, 99.99% trace metal), and 2 mg sodium ascorbate (Ph.Eur.).

^203/212^Pb in 5–10 mL 1.6 M HCl_suprapure_ was trapped on a custom-made Pb resin cartridge (100 mg PB-B10-F, Triskem, Bruz, France) preconditioned with 1 mL 2 M HCl_suprapure_. The captured activity was rinsed with 1 mL 2 M HCl_suprapure_. The activity was eluted with 2 mL NaAc/AcOH buffer (pH 6, 99.99% trace metal) directly into the reaction vial. The solution was heated at 95 °C for 15 min for ^203^Pb and 15 or 45 min for ^212^Pb. The reaction solution was then diluted with 4 mL of 0.9% NaCl solution and cooled.

Finally, the product was purified by using a C18 Plus light cartridge (WAT023501, Waters, Eschborn, Germany) preconditioned with 1 mL EtOH and 3 mL H_2_ O (wet condition). The cooled and diluted product solution (4 mL 0.9% NaCl) was slowly passed through the C18 cartridge. The C18 cartridge containing the product was rinsed with 2 mL of 0.9% NaCl solution and was directly eluted with 1 mL of 50% EtOH for injection directly through a vented sterile filter (0.22 µm, SLGVV255F, Millex-GV, Merck-Millipore, Darmstadt, Germany) into a product vial. Finally, the product was diluted with 7 mL of 0.9% NaCl solution.

Another purification method was performed using a Maxi-Clean (MC) SPE 0.5 mL IC-Chelate cartridge (5122565, S*Pure, Mainz, Germany) preconditioned with 5 mL H_2_ O_suprapure_ (wet condition). The cooled and diluted product solution (4 mL 0.9% NaCl) was slowly transferred through a vented sterile filter into the product vial via the MC cartridge and washed with an additional 2 mL 0.9% NaCl into the product vial.

### 4.2. Quality Control of Radiotracer

**Figure 3:**
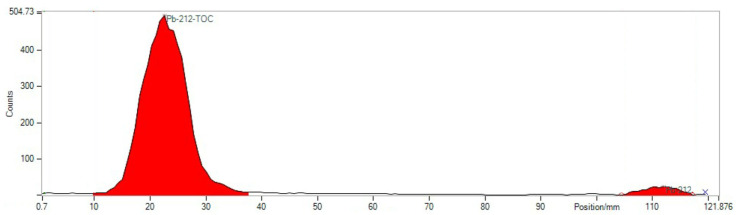
Corresponding TLC with Na-citrate as the eluent for the detection of unlabeled ^203/212^Pb at the front (position 115 mm, 3.9%) of the TLC. ^212^Pb-TOC runs at the origin (position 25 mm, 96.1%).

**Figure 4:**
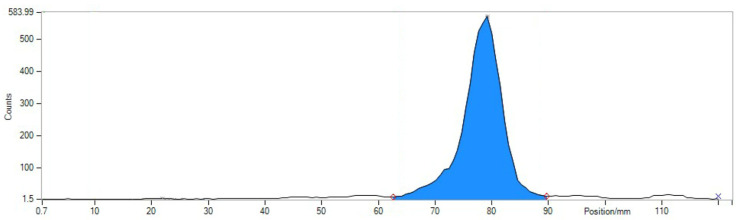
Corresponding TLC with NH_4_ Az:MeOH as the eluent for the detection of ^203/212^Pb-particles at the origin (position 20 mm, 0.6%) of the TLC. ^212^Pb-TOC runs at the front (position 80 mm, 99.4%.)

**Figure 5:**
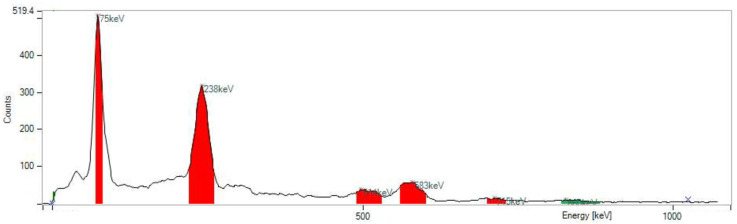
Corresponding gamma spectrum via MCA for verification of the RNP. Gamma lines found: ^212^Pb: 75, 238 keV; ^208^Tl: 510, 583, 860 keV; ^212^Bi: 727 keV.

Quality control included several standard tests established in the clinical manufacturing:

- TLC with eluent 0.1 M Na-citrate pH 5 (Start: ^203/212^Pb-PSC-TOC and particles (R*_f_* < 0.4), end: ^203/212^Pb-chloride) (Figure 3).
- TLC with eluent 1MNH_4_ Ac: MeOH1: 1 (Start: ^203/212^Pb-particles, end: ^203/212^Pb-PSC-TOC and ^203/212^Pb-chloride) (Figure 4).
- HPLC ^203/212^Pb-PSC-TOC *t*_R_ = 7.4 min. ^203/212^Pb-DOTA-TATE *t*_R_ = 7.1 min.
- pH value: 5.3 ± 0.5.
- RNP: ^212^Pb: 75 and 238 keV; ^212^Bi: 727 keV (6.7%); and ^208^Tl: 510 (22.6%), 583 (85.0%), and 860 (12.5%) keV (Figure 5).

### 4.3. Cell Uptake Experiments

Cell uptake of ^203/212^Pb-PSC-TOC was tested against our gold standard ^68^Ga-DOTATATE. AR42J (CRL-1492, ATCC^®^, Manassas, VA, USA), HEK293 sst2 (stably SST2 receptor transfected cells derived from Andrea Kliewer, University Hospital, Jena, Germany), and ZR75-1 (CRL-1500, ATCC^®^, Manassas, VA, USA); cells were seeded 1–2 days prior to the assay in 6-well plates to reach 0.5 × 10^6^ cells per well in a humidified atmosphere containing 5% CO_2_.

Each cell line was grown in its own medium:

- AR42J: Gibco RPMI 1640 medium (ATCC-Modification) supplemented with 10% fetal calf serum (FCS)
- HEK293 sst2 (stably transfected HEK293 cells): Dulbecco’s modified Eagle’s medium supplemented with 10% fetal calf serum (FCS), l-glutamine (2 mM = 1%), G-418 (50 mg/mL)
- ZR75-1: Gibco RPMI 1640 medium (w/o glutamine) supplemented with 10% fetal calf serum (FCS), 1% NEAA, 1 mM sodium pyruvate (1%), and 2 mM *N*-acetyl-alanyl-l-glutamine (1%)

After incubation with the respective radiotracer for 1 h, 24 h, 48 h, and 72 h, the medium, wash fraction (2 × 1 mL cold PBS 4 °C), and cell fraction (lysed with 1 mL 0.1 M NaOH and cell scraper) were collected, and the remaining activity in the fractions was measured with a gamma counter (HIDEX). The relative cell uptake in percent per 1 million cells was calculated between the medium, wash fraction, and lysate. Each cell incubation was performed in triplicate.

## 5. Conclusions

It was shown that the A_M_ of ^203/212^Pb-PSC-TOC has an effect on the cell uptake. No saturation was found in this work, but it is known from the literature that higher A_M_ > 100 MBq/nmol could have a negative effect on tumor uptake due to non-specific binding of the radioligand. It is noteworthy that higher A_M_ leads to higher cell uptake due to receptor-specific binding, and the values found in this work can be used as a reference for clinical application.

Another interesting radiochemical observation of this investigation is that the ^212^Pb labeling benefited from a longer reaction time and a larger amount of precursor to achieve a high RCY. Since ^203^Pb and ^212^Pb are elementally identical, the apparent differences must necessarily be due to the differences in stable Pb found in the solutions provided (generator eluant versus ^203^Pb solution), and thus, further investigation of the relative specific activity of the solutions is required.

In conclusion, for high tumor uptake, radiolabeling of 50 µg of PSC-TOC precursor should be performed with >1000 MBq ^203^Pb and with >500 MBq ^212^Pb.

## Figures and Tables

**Table 1:** ^203^Pb-PSC-TOC reactions.

Reactions ^1^	Starting Activity (MBq)	Product Activity (MBq)	RCY (%)	A_M_ (MBq/nmol)
1	74	72	97	2.3
2	174	128	74	5.4
3	40	36	90	1.3
4	302	249	82	7.9
5	536	488	91	15.3
6	455	438	96	13.7
7	2105	1972	94	61.6
8 ^2^	125	109	87	1.1
9	1301	1226	94	38.3

^1^ Reactions were always performed with 50 µg (31.7 nmol) VTM-α-NET for 15 min at 95 °C. ^2^ Exception: here, it is 156 µg (98.8 nmol) PSC-TOC.

**Table 2:** ^212^Pb-PSC-TOC reactions.

Reactions ^1^	Starting Activity (MBq)	Product Activity (MBq)	RCY (%)	A_M_ (MBq/nmol)
1	24	10	40	0.8
2	4	2	53	0.2
3	490 *	176 *	36	13.9
4	214 *	48 *	22	3.8
5 ^2^	24	11	48	0.8

^1^ Reactions were always performed with 20 µg (12.7 nmol) VTM-α-NET for 15 min at 95 °C. ^2^ Exception: here, it is 20 µg (13.7 nmol) DOTA-TATE. * Normalization was performed (see Table 4).

**Table 3:** Optimized ^212^Pb-PSC-TOC reactions.

Reactions ^1^	Starting Activity (MBq)	Product Activity (MBq)	RCY (%)	A_M_ (MBq/nmol)
1	522 *	490 *	98	15.5
2	328	315	97	9.9
3	274	260	99	8.2
4	177	165 *	93	5.2
5	146	104 *	71	3.3
6	95	78 *	82	2.5
7	32	31	99	1.0

^1^ Reactions were always performed with 50 µg (31.7 nmol) VTM-α-NET for 45 min at 95 °C. * Normalization was performed (see Table 4).

**Table 4:** Normalization factors for the activity measurements of ^212^Pb after separation from the daughter nuclides by Pb resin for the ISOMED 2010 dose calibrator.

Time after Separation (min)	Normalization Factor
0	1.86
5	1.80
30	1.58
60	1.37
90	1.26
120	1.18

**Table 5:** Cell uptake statistics for ^203^Pb-PSC-TOC.

Cells	Incubation Time (h)	Molar Activity (MBq/nmol)							
		1.1	1.3	2.3	5.4	7.9	13.7	15.3	38.3
AR42J uptake (%)	1	0.4 ± 0.1				4.1 ± 0.2	4.2 ± 0.0	2.6 ± 0.2	7.5 ± 0.4
	24	0.6 ± 0.0				8.6 ± 0.5	9.1 ± 0.4	4.9 ± 0.6	13.6 ± 0.2
	48								17.9 ± 0.7
	72								16.5 ± 1.3
HEK293 sst2 uptake (%)	1		1.2 ± 0.3	1.3 ± 0.1	3.9 ± 0.2	1.6 ± 0.3	2.1 ± 0.1	2.8 ± 0.1	1.2 ± 0.3
	24		2.6 ± 0.6	1.0 ± 0.0	2.8 ± 0.2	4.0 ± 0.3	4.3 ± 0.2	4.3 ± 0.9	2.6 ± 0.6

**Table 6:** Cell uptake statistics for ^212^Pb-PSC-TOC.

Cells	Incubation Time (h)	Molar Activity (MBq/nmol)							
		0.2	0.8	1.0	3.3	3.8	5.2	13.9	15.5
AR42J uptake (%)	1	0.8 ± 0.1	1.4 ± 0.1	1.5 ± 0.1		n.d.	3.8 ± 0.2	5.3 ± 0.3	4.6 ± 0.3
	24	2.6 ± 0.2	4.4 ± 0.4	4.5 ± 0.0	8.0 ± 1.0	1.5 ± 0.1	8.7 ± 0.7		17.0 ± 0.7
	48	3.2 ± 0.4				3.1 ± 0.1	9.6 ± 0.7		
	72							5.2 ± 0.4	25.0 ± 0.5
HEK293 sst2 uptake (%)	1							5.1 ± 0.5	
	24					4.0 ± 0.5			
	48					9.1 ± 0.5			
	72							7.8 ± 0.0	

## Data Availability

All data can be referred to on request to the corresponding author.
